# Health risk assessment of potentially toxic elements in public pipe-borne water from selected households in Abeokuta, Southwestern, Nigeria

**DOI:** 10.1016/j.toxrep.2025.101970

**Published:** 2025-02-20

**Authors:** Adewale M. Taiwo, Adediran O. Coker

**Affiliations:** Department of Environmental Management and Toxicology, Federal University of Agriculture, Abeokuta, Ogun State PMB 2240, Nigeria

**Keywords:** Water quality, Cancer risk, Non-carcinogenic effects, Inorganic contaminants, Permissible limit

## Abstract

Public pipe-borne water serves as a significant source of potable water in some communities in Abeokuta. However, the presence of potentially toxic elements (PTEs) at certain concentrations in drinking water can render it unsuitable for consumption due to associated health risks. This study assessed the health risks posed by PTEs in public pipe-borne water collected from ten households in six communities in Abeokuta, Southwestern Nigeria. Pipe-borne water samples were subjected to PTE (lead (Pb), cadmium (Cd), chromium (Cr), copper (Cu), and zinc (Zn) analysis using an Atomic Absorption Spectrophotometer, following standard digestion procedures for the water samples. The collected data were analyzed using descriptive and inferential statistics. The health risk assessment was conducted for the Hazard Quotient (HQ) and Cancer Risk (CR) using the USEPA IRIS models. The water quality index (WQI) was also evaluated. Results indicated varying levels of PTEs, with certain areas exceeding the permissible limits for Cd (0.01–0.02 mg/L), Cr (0.05–0.11 mg/L), and Pb (0.50–0.97 mg/L), posing significant health risks to humans. The health risk assessment revealed that the HQ for Cd, Cr, and Pb exceeded the permissible limit of 1.0 in pipe-borne water consumed by children and infants, indicating potential adverse effects. Additionally, the CR values for Cd, Cr, and Pb in water samples consumed by all age groups also surpassed the permissible limit of 1.0 × 10^−4^, suggesting a probable risk of cancer development. The WQIs exceeding the threshold limit of 300 were observed in pipe-borne water samples, indicating that the water is unfit for consumption. Therefore, this study recommends the removal of potential toxic elements at the point of collection by using taps fitted with activated carbon filters.

## Highlights

• Health risks posed by potentially toxic elements in public pipe-borne water were assessed.
• Concentrations of Cd, Cr, and Pb in pipe-borne water exceeded the acceptable limit.
• The risk assessment data revealed HQ > 1.0 for Cd, Cr, and Pb, indicating adverse health effects.
• The CR values for Cd, Cr, and Pb in water samples were also the permissible limit.

## Graphical Abstract

**Figure:**
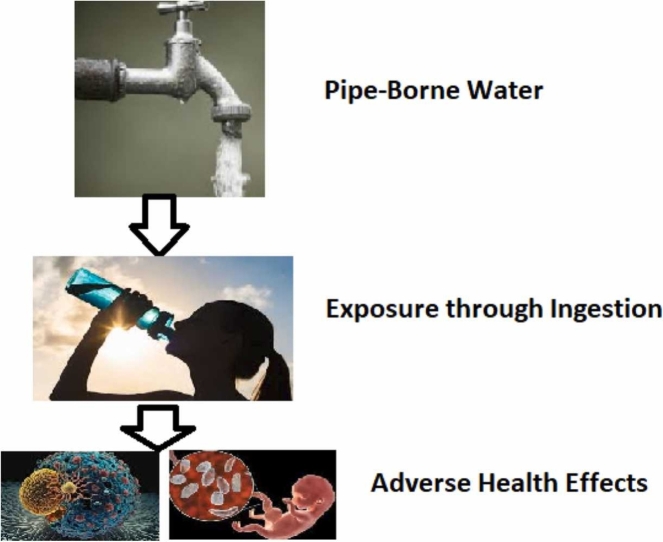

## 1 Introduction

Water quality is a critical factor in public health, particularly in regions where access to clean and safe drinking water is limited. The necessity of water for quenching thirst, maintaining hygiene, and providing food is indispensable for human survival. Globally, rapid economic growth, population explosion, climate change, and urbanization have led to an increased demand for water for domestic and drinking purposes [44], [6]. Unfortunately, more than 884 million people worldwide still lack access to potable drinking water [7]. In Nigeria, at least 52 % of the population does not have access to safe drinking water [26].

Potable water is essential for the health and survival of humans. However, drinking water can become contaminated due to various human activities, including industrialization, agriculture, urbanization, vehicular emissions, and improper waste disposal. Additionally, natural phenomena such as siltation, saltwater intrusion, weathering of bedrock, and the presence of radioisotopes can also contribute to contamination [23], [33]. Inorganic chemical contaminants such as potentially toxic elements (PTEs) can lead to numerous health issues, including damage to vital organs (such as the kidneys, liver, and lungs), impairment of the central and peripheral nervous systems, cancer, DNA mutations, and birth defects [15], [18], [36]. In developing countries, exposure to unsafe drinking water results in more than one million deaths annually [29].

Pipe-borne water is a vital source of drinking water globally, particularly in urban areas. It is often regarded as a safer and more reliable option compared to groundwater and surface water, which are susceptible to contamination from both natural and human activities. PTEs such as lead, copper, cadmium, arsenic, and mercury can contaminate water supplies from various sources. Corrosion of household plumbing fixtures, fittings, pipes, and solder can leach metals like lead and copper into public water supplies [16]. Cadmium contamination in piped water may occur due to impurities in the zinc used in galvanized pipes or fittings [8]. For example, Dabo and Saleh [9] reported that the concentrations of chromium (Cr) and lead (Pb) in tap water from Kano exceeded the acceptable standards set by the World Health Organization (WHO) by 14 and 27 times, respectively. Additionally, a study conducted by Nwaedozie et al. [21] revealed that 30 % of the pipe-borne water sampled in selected communities in Abuja contained aluminum (Al) and iron (Fe) concentrations that surpassed the permissible limits established by the WHO [40].

PTEs could pose significant risks to drinking water due to their inherent health hazards for consumers. Consequently, health risk assessments of drinking water are essential for understanding the potential impacts of contaminants on human health [35], [34]. Unfortunately, the levels and health risk assessment of potentially toxic elements (PTEs) in pipe-borne water in Abeokuta, southwestern Nigeria, are rarely reported. The few available studies on pipe-borne water in Nigeria have primarily focused on the physicochemical and microbiological characteristics of this water [17], [20], [21], [25], [9]. This study aims to assess the concentrations of heavy metals and the associated health risks linked to the consumption of public piped water in Abeokuta, Nigeria.

## 2 Materials and methods

### 2.1 Study area

Abeokuta, the largest city and capital of Ogun State in southwestern Nigeria, is situated between longitudes 7° 09′ E and 7° 39′ E, and latitudes 3° 20′ N and 3° 54′ N. This historic city is located on the eastern bank of the Ogun River, surrounded by a series of rocky outcroppings and wooded savanna. The metropolis of Abeokuta encompasses four Local Government Areas: Abeokuta South, Abeokuta North, and parts of Odeda and Obafemi Owode. The city covers an area of approximately 2320 km², which is expanding in all directions due to rapid population growth and urbanization [24].

Water samples were collected from ten households in six different communities: Lantoro (Abeokuta South LGA), Alogi (Odeda LGA), Temidire (Abeokuta North LGA), Olorunsogo (Abeokuta South LGA), Gbonagun (Odeda LGA), and Ajegunle (Abeokuta North LGA) between June and July, 2024. These communities were selected to represent various socio-economic and geographical areas. They are residential neighborhoods supplied with potable water from the Ogun State Water Corporation plant at the Oke Temidire reservoir.

### 2.2 Sample collection and laboratory analysis

Sixty (60) water samples were collected from six residential households, with ten (10) samples taken from each community: Lantoro, Alogi, Temidire, Olorunsogo, Gbonagun, and Ajegunle. The samples were collected in clean, sterilized 750 mL bottles and acidified with nitric acid (HNO_3_) in accordance with the methods outlined by Sharma and Tyagi [31]. Standard protocols were followed to prevent contamination. Each sample was labeled with a code corresponding to its community: LAN for Lantoro, ALO for Alogi, TEMI for Temidire, OLO for Olorunsogo, GBON for Gbonagun, and AJE for Ajegunle.

Temperature, pH, total dissolved solids (TDS), electrical conductivity (EC) and Oxidation Reduction Potential (ORP) were analysed electronically using a multifunction water quality tester (BLE-P-3, Fuzhou Hedao Trade Co., Ltd., Taijiang District, Fuzhou, Fujian, China) after calibrating the instrument with standard buffer solutions of pH 4, 7 and 9. The multifunction water quality tester electrode was thoroughly rinsed with distilled water before measuring the temperature, pH, EC, TDS and ORP of the water samples in situ at the point of collection. The samples were then transported to the laboratory under controlled conditions.

Samples for metal analysis (100 mL) were digested with 10 mL of concentrated nitric (HNO_3_) on a hotplate in a fume hood for 30 minutes. The digested samples were then filtered using Whatman filters with a pore size of 1.0 µm and analyzed for heavy metals, including zinc (Zn), copper (Cu), chromium (Cr), cadmium (Cd), and lead (Pb), using atomic absorption spectrophotometry (AAS) with a Bulk Scientific (210/211VGp, Connecticut, USA). Quality control measures, such as the use of standard reference materials and duplicate samples, were implemented to ensure accuracy.

### 2.3 Data analysis

Laboratory-obtained data were analyzed using descriptive statistics (mean and standard deviation) and inferential statistics (Duncan Multiple Range Test) with the Statistical Package for the Social Sciences (SPSS) for Windows (version 23.0).

### 2.4 Health risk assessment

The health risk assessment of metals in pipe-borne water was evaluated for both carcinogenic (cancer risk) and non-carcinogenic (hazard quotient and hazard index) adverse effects by utilizing the models established by the United States Environmental Protection Agency Integrated Risk Information System [39].

(1) $$\mathrm{Estimated Daily Dose}(\mathrm{EDD})\;=\;\frac{\mathrm{C x IR x EF x ED}}{\mathrm{BW x AT}}$$

(2) $$\mathrm{Hazard quotient}(\mathrm{HQ})\;=\;\frac{\mathrm{EDD}}{\mathrm{RfD}}$$

(3) $$\mathrm{Hazard index}(\mathrm{HI})\;=\;\sum_{i=1}^{n}\mathrm{HQ}i=1\ldots n$$

(4) Cancer Risk (CR) = EDD x CSF

Where, EDD (mg kg^−1^ day^−1^) = Estimated daily dose of potentially toxic elements (PTEs) exposed through ingestion of pipe-borne water, C = Concentration of PTEs observed in pipe-borne water (mg L^−1^), IR = Ingestion rate of pipe-borne water [2, 1, and 0.75 L/day for adults, children and infants, respectively [41]], EF = Exposure frequency (350 days year^−1^; Taiwo *et al.,* 2021), ED = 30, 6 and 1 year for carcinogenic effects in adults, children and infants, respectively [38], AT = Averaging time or life expectancy; AT = ED for non-carcinogenic effects, while AT = 61.33, 6 and 1 × 365 days for carcinogenic effect in adults, children and infants, respectively [32], BW = Body weight [60, 15 and 10 kg for adults, children and infants, respectively [41])], n = numbers of elements observed, CSF = Cancer slope factor of PTEs.

HQ > 1.0 shows non-carcinogenic adverse health effects; HQ < 1.0 indicates no deleterious health effects; CR > 1.0 × 10^−4^ indicates possible development of cancer; while CR < 1.0 × 10^−4^ suggests no cancer risk.

### 2.5 Water quality index (WQI) analysis

Water Quality Index (WQI) analysis was conducted to assess the suitability of groundwater for drinking purposes concerning potentially toxic elements (PTE) pollution. This analysis reflects the cumulative impact of various PTEs. The method employs parameters such as the water quality rating scale, relative weight, and sub-index WQI to compute the overall WQI (Eq. 5 to 8, [10]). The WQI was determined based on five PTEs: zinc (Zn), copper (Cu), chromium (Cr), cadmium (Cd), and lead (Pb). Detailed information regarding the WQI of these PTEs is provided in Table S1 (in the supplementary material).

Water quality rating scale, qi was estimated as:

(5) $$\mathrm{Qi}=\;\frac{\mathrm{Cn}}{\mathrm{Sn}}\times 100$$

Where C_n_ = Concentration of each (n^th^) parameter, S_n_ = standard value of each (n^th^) parameter.

Relative weight, W_n_ was calculated as:

(6) $$Wn=\;\frac{\mathrm{Wn}}{\sum_{n=1}^{y}\mathrm{Wn}}$$

Where w_n_ =weight of each (n^th^) parameter and y = number of parameters.

The overall WQI was estimated:

(7) SI_n_=W_n_ x Q_n_

Where SI_n_ = sub-index of n^th^ parameter, Q_n_ = Water quality rating scale, and W_n_ = Relative weight.

(8) WQI = ∑SI_n_

WQI value less than 50 shows an excellent water quality; WQI range of 50.1–100 indicates good water quality; WQI variation of 100.1–200 established poor water quality; WQI range of 200.1–300 confirms very poor water quality; while WQI greater than 300 reveals water unfit for drinking [27].

## 3 Results and discussion

### 3.1 Physico-chemical and potentially toxic elements in pipe-borne water samples

Table 1 presents the physicochemical parameters of pipe-borne water samples collected from households in the Lantoro, Alogi, Temidire, Olorunsogo, Gbonagun, and Ajegunle communities in Abeokuta. The mean temperature of the pipe-borne water samples ranged from 28.3 ± 3.00°C to 28.3 ± 1.44°C. There were minimal temperature variations in the pipe-borne water samples; however, Ajegunle and Alogi exhibited statistically significant (p < 0.05) higher temperatures compared to the other four sampled communities. The mean temperature observed in the pipe-borne water samples across all communities slightly exceeded the typical water temperature of 27°C found in tropical regions [5].

**Table 1:** Physico-Chemical Parameters of Pipe-borne water.

		N	Mean	Std. Deviation	Minimum	Maximum	WHO [40]
Temperature (°C)	LAN	10	28.3a	3.00	19.9	29.9	
	ALO	10	32.5c	1.03	31.2	34.3	
	TEMI	10	30.0b	1.59	27.8	33.1	
	OLO	10	30.0b	0.95	28.4	32.0	
	GBON	10	30.9b	0.88	30.1	33.0	
	AJE	10	32.8c	1.44	31.0	35.4	
	Total	60	30.8	2.23	19.9	35.4	
pH	LAN	10	7.01a	0.19	6.63	7.25	6.5–8.5
	ALO	10	7.30b	0.11	7.10	7.45	
	TEMI	10	7.23b	0.18	6.87	7.50	
	OLO	10	7.00a	0.22	6.55	7.34	
	GBON	10	6.99a	0.24	6.60	7.29	
	AJE	10	7.09ab	0.09	6.99	7.29	
	Total	60	7.10	0.21	6.55	7.50	
ORP	LAN	10	125c	9.1	111.3	143.3	
(mV)	ALO	10	81.6a	8.4	64.0	90.0	
	TEMI	10	99.3b	12.5	78.3	117.3	
	OLO	10	94.7b	18.6	59.5	117.3	
	GBON	10	79.2a	6.3	70.7	87.7	
	AJE	10	121c	8.0	108.3	134.0	
	Total	60	100.3	20.9	59.5	143.3	
EC	LAN	10	230bc	16.3	217	262	
(µS/cm)	ALO	10	220abc	3.50	210	223	
	TEMI	10	216a	3.92	208	223	
	OLO	10	219abc	2.94	213	222	
	GBON	10	231b	26.8	213	282	
	AJE	10	218ab	4.95	212	229	
	Total	60	222	14.0	208	282	
TDS	LAN	10	115b	8.29	109	131	600
(mg/L)	ALO	10	110ab	1.60	106	111	
	TEMI	10	108ab	1.71	106	112	
	OLO	10	105a	12.2	72.3	111	
	GBON	10	115b	12.7	107	141	
	AJE	10	109ab	2.36	106	114	
	Total	60	110	8.60	72.3	141	

EC- Electrical conductivity, TDS – Total dissolved solids, ORP-Oxidation Reduction Potential, LAN- Lantoro, ALO- Alogi, TEMI- Temidire, OLO- Olorunsogo, GBON- Gbonagun, AJE- Ajegunle., Means with similar alphabets along the column are not significantly different at p > 0.05 according to the Duncan Multiple Range Test

The mean pH value ranged from 6.99 ± 0.24 in the Ajegunle community to 7.3 ± 0.11 in the Alogi community. The average pH of water samples from the six communities fell within the World Health Organization's (WHO) permissible range of 6.5–8.5 for drinking water [40]. These findings indicate that the pH of the pipe-borne water varied from slightly acidic to slightly alkaline, making it suitable for drinking purposes, as the human body typically maintains a pH range of 7.0–7.2, which is essential for sustaining and protecting human health [30]. The pH values of the pipe-borne water samples collected in this study are comparable to the average pH levels of 6.77 and 7.03–7.23 reported for pipe-borne water in Jalingo, Taraba [13] and Garki, Abuja [20], Nigeria, respectively.

The mean value of the Redox (Reduction-Oxidation) potential for Gbonagun was the lowest at 79.2 ± 6.3 mV, while the highest value of 121 ± 8.0 mV was recorded in the Ajegunle community. There was a significant variance in the mean Redox potential values across the six communities. The lowest electrical conductivity (EC) value, measured at 216 ± 3.92 μS/cm, was observed in Temidire, whereas the highest mean value of 230 ± 16.3 μS/cm was found in Lantoro. The mean electrical conductivity (EC) values observed at various locations within the study area were within the permissible limit of 1000 μS/cm for drinking water [42]. The study conducted by Hassan et al. [13] reported lower EC concentration of 155 μS/cm in pipe-borne water from Jalingo, Taraba, Nigeria. In contrast, higher levels were documented in the pipe-borne water samples from Abuja, Nigeria [4]. The total dissolved solids (TDS) measured in the sampled communities were also within the permissible limit of 600 mg/L [40], with mean values ranging from 105 ± 12.12 mg/L at Olorunsogo to 115 ± 12.7 mg/L at Gbonagun.

Table 2 presents the concentrations of potentially toxic elements (PTEs) in pipe-borne water samples. Zinc (Zn) concentrations ranged from 0.23 ± 0.20 mg/L in the Lantoro community to 0.65 ± 0.34 mg/L in the Gbonagun community. The mean concentrations of zinc in all water samples were below the permissible limit of 5.0 mg/L for drinking water, as established by the World Health Organization [40]. The mean concentrations of copper (Cu) varied from 0.03 ± 0.03 mg/L in Lantoro to 0.13 ± 0.06 mg/L in Alogi. The mean concentrations of copper in the study area remained within the WHO's threshold limit of 2 mg/L for drinking water [40].

**Table 2:** PTM levels in water samples.

		N	Mean	Std. Deviation	Minimum	Maximum	WHO [40]
Zn	LAN	7	0.23a	0.20	0.08	0.63	5.0
(mg/L)	ALO	7	0.35ab	0.21	0.08	0.77	
	TEMI	8	0.27ab	0.19	0.10	0.69	
	OLO	8	0.26ab	0.22	0.07	0.63	
	GBON	10	0.65c	0.34	0.25	1.20	
	AJE	10	0.50bc	0.26	0.17	0.96	
	Total	50	0.40	0.29	0.07	1.20	
Cu	LAN	10	0.03a	0.03	< 0.01	0.09	2.0
	ALO	10	0.05a	0.05	< 0.01	0.14	
(mg/L)	TEMI	10	0.06a	0.05	< 0.01	0.18	
	OLO	10	0.07ab	0.05	< 0.01	0.18	
	GBON	10	0.13b	0.06	0.04	0.21	
	AJE	10	0.12b	0.07	0.04	0.28	
	Total	60	0.08	0.06	0.00	0.28	
Cr	LAN	10	0.05ab	0.02	0.02	0.09	0.05
(mg/L)	ALO	10	0.06ab	0.03	0.02	0.11	
	TEMI	10	0.10ab	0.09	< 0.01	0.22	
	OLO	10	0.05a	0.03	< 0.01	0.10	
	GBON	10	0.10ab	0.05	0.03	0.17	
	AJE	10	0.11b	0.10	0.03	0.32	
	Total	60	0.08	0.06	< 0.01	0.32	
Cd	LAN	10	0.02a	0.01	< 0.001	0.04	0.005
	ALO	10	0.02a	0.01	< 0.001	0.04	
(mg/L)	TEMI	10	0.02a	0.01	< 0.001	0.04	
	OLO	10	0.02a	0.01	< 0.001	0.04	
	GBON	10	0.03a	0.01	< 0.001	0.04	
	AJE	10	0.02a	0.01	< 0.001	0.04	
	Total	60	0.02	0.01	< 0.001	0.04	
Pb	LAN	10	0.50a	0.16	0.33	0.76	0.05
	ALO	10	0.60a	0.16	0.38	0.84	
(mg/L)	TEMI	10	0.55a	0.26	0.20	0.90	
	OLO	10	0.68a	0.26	0.33	1.05	
	GBON	10	0.55a	0.13	0.31	0.74	
	AJE	10	0.97b	0.46	0.28	1.53	
	Total	60	0.64	0.30	0.20	1.53	

LAN- Lantoro, ALO- Alogi, TEMI- Temidire, OLO- Olorunsogo, GBON- Gbonagun, AJE- Ajegunle., Means with similar alphabets along the column are not significantly different at p > 0.05 according to the Duncan Multiple Range Test

The concentrations of chromium (Cr) ranged from 0.05 ± 0.03 mg/L in Olorunsogo and Lantoro to 0.11 ± 0.10 mg/L in Ajegunle. The Cr levels detected in the pipe-borne water from the six sampled communities exceeded the acceptable limit of 0.05 mg/L set by the World Health Organization [40], with the exception of Olorunsogo and Lantoro. The average concentration of Cr in the pipe-borne water within the study area surpassed the permissible limit of 0.05 mg/L. The health implications of chromium have been extensively discussed in the works of Jarup [14] and Taiwo et al. [34]. Chromium exposure can lead to both carcinogenic and non-carcinogenic adverse health effects.

There was no statistical variance in the cadmium (Cd) values obtained from the six sampled communities, with the highest mean concentration recorded at 0.03 ± 0.01 mg/L in Gbonagun. These Cd concentrations exceeded the World Health Organization's (WHO) threshold limit of 0.005 mg/L for safe drinking water [40]. Continuous exposure to cadmium (Cd) in these water samples can lead to irreversible and harmful health effects in consumers. The values obtained for lead (Pb) in the water samples showed no statistical variance among the sampling sites, except at the Ajegunle community, which exhibited the highest mean concentration of 0.97 ± 0.46 mg/L. The concentrations of Pb in pipe-borne water at each sampling location indicated mean values that were ten times higher than the World Health Organization (WHO) permissible limit of 0.05 mg/L ([40]. The presence of elevated levels of lead in pipe-borne water may be attributed to the corrosion of lead-soldered joints [19]. The high concentration of lead in the water poses a significant health risk to the public, particularly to children and infants.Lead is a well-known pediatric toxic metal associated with numerous health challenges, ranging from impaired intelligence quotient to cancer in children and infants [36].

Unlike the present study, which observed extremely high levels of Pb in pipe-borne water, the previous study by Nwaedozie et al. [21] reported Pb and Cd concentrations below the detection limit in pipe-borne water from Abuja. However, Al and Fe were identified as metals posing significant health challenges in the pipe-borne water. Similarly, the lead levels detected in pipe-borne water from Dutse, Jigawa, Nigeria, were alarmingly high, exceeding the WHO permissible limit by 9–27 times [1]. Furthermore, elevated concentration ranges of cadmium, chromium and lead (0.08–0.10, 0.04–0.22, and 0.07–0.36 mg/L, respectively) were observed in treated water at the Eleyele treatment facility in Ibadan [3]. The average levels of Cu (3.02 mg/L), Zn (12 mg/L), Cd (1.43 mg/L), Pb (2.79 mg/L) and Cr (5 mg/L) observed in the pipe-borne water from Ile Ife exceeded the values reported in this study. These values surpassed the permissible limits for the respective metals.

### 3.2 Health risk assessment

#### 3.2.1 Estimated daily metal intake from pipe-borne water

Table S2 (in the supplementary materials) presents the estimated daily dose (EDD) of potentially toxic elements (PTEs) ingested by adults, children, and infants across various communities. The EDD is a crucial metric used in risk assessment to estimate the amount of a chemical (in this case, metals) that an individual consumes daily relative to their body weight. The EDD of zinc (Zn) for adults ranged from 0.0051 to 0.021 mg/kg/day. Generally, the estimated daily intake of Zn by children was higher than that of adults, with EDD values ranging from 0.01 mg/kg/day (Lantoro) to 0.042 mg/kg/day (Gbonagun). However, infants exhibited the highest EDD, varying from 0.011 to 0.047 mg/kg/day.

The EDD for copper (Cu) ranged from 0.00086 to 0.0041 mg/kg/day in adults, 0.0017–0.0082 mg/kg/day in children, and 0.0019–0.0092 mg/kg/day in infants. The EDD for chromium (Cr) also varied, ranging from 0.0015 to 0.0034 mg/kg/day in adults, 0.003–0.0067 mg/kg/day in children, and 0.0034–0.0076 mg/kg/day in infants. Cadmium (Cd) had EDD values ranging from 0.00051 to 0.00086 mg/kg/day in adults, 0.001–0.0017 mg/kg/day in children, and 0.0012–0.0019 mg/kg/day in infants. The EDD of lead (Pb) varied from 0.016 to 0.031 mg/kg/day in adults, 0.032–0.062 mg/kg/day in children, and 0.036–0.07 mg/kg/day in infants.

#### 3.2.2 Hazard quotients (HQ) of PTEs

Table 3 presents the Hazard Quotient (HQ) values of potential toxic metals (PTEs) in pipe-borne water across various locations within the study area. The HQ serves as a risk assessment tool designed to estimate the potential non-carcinogenic health risks associated with exposure to hazardous substances.

**Table 3:** Hazard Quotient Values of Metals in Pipe-borne water.

				Adults				Children				Infants	
		Mean	Std. Deviation	Minimum	Maximum	Mean	Std. Deviation	Minimum	Maximum	Mean	Std. Deviation	Minimum	Maximum
Zn	LAN	0.017	0.021	0.000	0.067	0.034	0.042	0.000	0.134	0.038	0.047	0.000	0.151
	ALO	0.026	0.026	0.000	0.082	0.052	0.052	0.000	0.164	0.059	0.058	0.000	0.185
	TEMI	0.023	0.022	0.000	0.074	0.046	0.043	0.000	0.147	0.052	0.048	0.000	0.165
	OLO	0.022	0.024	0.000	0.067	0.045	0.048	0.000	0.134	0.050	0.054	0.000	0.151
	GBON	0.070	0.036	0.027	0.128	0.139	0.073	0.053	0.256	0.157	0.082	0.060	0.288
	AJE	0.054	0.028	0.018	0.102	0.107	0.056	0.036	0.205	0.121	0.063	0.041	0.230
Cu	LAN	0.022	0.026	0.000	0.072	0.043	0.052	0.000	0.144	0.049	0.059	0.000	0.162
	ALO	0.035	0.044	0.000	0.112	0.070	0.088	0.000	0.224	0.079	0.099	0.000	0.252
	TEMI	0.039	0.043	0.000	0.144	0.078	0.085	0.000	0.288	0.088	0.096	0.000	0.324
	OLO	0.058	0.043	0.008	0.144	0.117	0.085	0.016	0.288	0.131	0.096	0.018	0.324
	GBON	0.102	0.050	0.032	0.168	0.205	0.100	0.064	0.336	0.230	0.112	0.072	0.378
	AJE	0.093	0.057	0.032	0.224	0.185	0.114	0.064	0.447	0.209	0.128	0.072	0.503
Cr	LAN	0.485	0.243	0.206	0.928	0.969	0.487	0.412	1.856	1.090	0.547	0.464	2.088
	ALO	0.639	0.303	0.206	1.134	1.279	0.606	0.412	2.268	1.438	0.681	0.464	2.552
	TEMI	1.083	0.915	0.103	2.268	2.165	1.830	0.206	4.537	2.436	2.058	0.232	5.104
	OLO	0.474	0.292	0.103	1.031	0.949	0.585	0.206	2.062	1.067	0.658	0.232	2.320
	GBON	1.041	0.555	0.309	1.753	2.083	1.110	0.619	3.506	2.343	1.249	0.696	3.944
	AJE	0.794	0.999	0.000	3.299	1.588	1.997	0.000	6.599	1.786	2.247	0.000	7.424
Cd	LAN	0.575	0.330	0.320	1.279	1.151	0.660	0.639	2.557	1.295	0.743	0.719	2.877
	ALO	0.735	0.400	0.320	1.279	1.470	0.800	0.639	2.557	1.654	0.900	0.719	2.877
	TEMI	0.511	0.431	0.000	1.279	1.023	0.863	0.000	2.557	1.151	0.971	0.000	2.877
	OLO	0.511	0.457	0.000	1.279	1.023	0.914	0.000	2.557	1.151	1.028	0.000	2.877
	GBON	0.863	0.303	0.320	1.279	1.726	0.606	0.639	2.557	1.942	0.682	0.719	2.877
	AJE	0.543	0.428	0.000	1.279	1.087	0.855	0.000	2.557	1.223	0.962	0.000	2.877
Pb	LAN	4.566	1.433	3.014	6.941	9.132	2.866	6.027	13.881	10.274	3.224	6.781	15.616
	ALO	5.516	1.472	3.470	7.671	11.032	2.944	6.941	15.342	12.411	3.312	7.808	17.260
	TEMI	5.023	2.379	1.826	8.219	10.046	4.759	3.653	16.438	11.301	5.354	4.110	18.493
	OLO	6.219	2.400	3.014	9.589	12.438	4.800	6.027	19.178	13.993	5.400	6.781	21.575
	GBON	5.050	1.235	2.831	6.758	10.100	2.470	5.662	13.516	11.363	2.779	6.370	15.205
	AJE	8.858	4.183	2.557	13.973	17.717	8.366	5.114	27.945	19.931	9.412	5.753	31.438

LAN- Lantoro, ALO- Alogi, TEMI- Temidire, OLO- Olorunsogo, GBON- Gbonagun, AJE- Ajegunle.

The hazard quotient (HQ) values for zinc (Zn) across all locations and demographic groups were below the permissible limit of 1.0, indicating no significant risk. Similarly, the HQ values for copper (Cu) were also below the permissible limit of 1.0, suggesting no adverse health effects. Cr exhibited hazard quotient (HQ) values exceeding the threshold limit of 1.0 for infants at all sampling sites, for children at two-thirds of the sites, and for adults at one-third of the locations. Conversely, Cd demonstrated HQ values greater than 1.0 for infants and children at all sampling sites, while it showed HQ values less than 1.0 for adults at all monitoring stations. The HQ data for Cd indicated serious adverse effects for infants and children.

The hazard quotients (HQs) for lead (Pb) were generally higher than the permissible limit of 1.0 across all demographic categories. However, infants consistently exhibited the highest HQ values for all metals and locations, indicating that they are the most vulnerable group to the potential health risks associated with consuming pipe-borne water.

Pipe-borne water samples from Ajegunle consistently displayed the highest HQ values for all tested metals, particularly lead, highlighting a severe health risk that necessitates urgent intervention. In contrast, pipe-borne water samples from Lantoro showed the lowest HQ values for most metals, suggesting a relatively lower risk.

Taiwo et al. [37] similarly observed the HQ greater than 1.0 for Cd and Cr in sachet water from Ogun state, Nigeria. The previous study by Adeniyi et al. [2] demonstrated a HQ greater than 1.0 in borehole water samples collected from selected households in southwestern Nigeria. Similarly, Taiwo et al. [37] observed that the HQ and HI values for Cr and Cd exceeded the permissible limit of 1.0 in sachet water samples collected from the Abeokuta metropolis.

Studies from Ghana, South Africa and Ethopia had documented HQ > 1.0 for Pb in potable water [11], [12], [22]. The present non-carcinogenic health risk data indicate that regular monitoring, public health interventions, and potentially water treatment measures are essential to mitigate these risks, especially in the most affected areas.

**Fig. 1:**
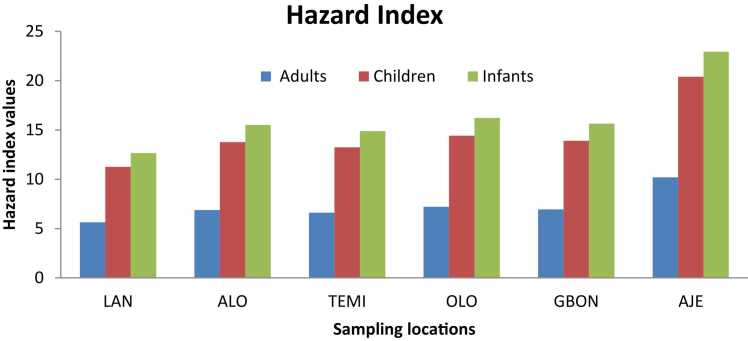
Hazard Index Values of Metals in Pipe-borne water. LAN- Lantoro, ALO- Alogi, TEMI- Temidire, OLO- Olorunsogo, GBON- Gbonagun, AJE- Ajegunle.

Fig. 1 illustrates the hazard index values of metals in pipe-borne water samples collected from six communities. The Hazard Index (HI) is a crucial metric used in health risk assessments to evaluate the potential non-carcinogenic health risks associated with exposure to multiple chemicals. It is calculated by summing the hazard quotients (HQs) of various contaminants. An HI value greater than 1.0 indicates a potential health risk related to exposure. HI values exceeding the permissible limit of 1.0 were documented at each sampling site, further highlighting the non-carcinogenic adverse health effects of potentially toxic elements in the water samples.

The Hazard Index (HI) values for all sites are significantly greater than 1.0 across all population groups, including adults, children, and infants. This indicates a potential risk of non-carcinogenic effects for individuals exposed to these pipe-borne water sources. Notably, infants exhibited the highest HI values across all locations, suggesting that they are the most vulnerable to potential health risks due to their lower body weight and higher water intake relative to their size.

The sampling site in Ajegunle consistently recorded the highest health index (HI) values across all population groups, while the Lantoro community exhibited the lowest HI values, which remained above the safety threshold of 1.0.

**Fig. 2:**
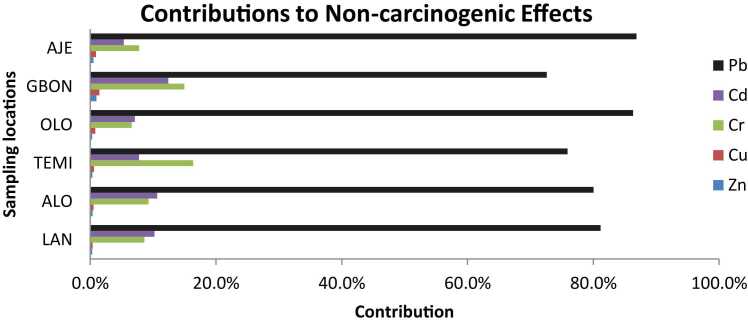
Contributions by Metals to Non-Carcinogenic Effects. LAN- Lantoro, ALO- Alogi, TEMI- Temidire, OLO- Olorunsogo, GBON- Gbonagun, AJE- Ajegunle.

Fig. 2 presents the contributions to non-carcinogenic effects, with Pb accounting for the highest percentage across all sites, ranging from 72 % to 86 %. Cadmium (Cd) follows, with contributions varying from 5 % to 12 %.

#### 3.2.3 Cancer risk values of PTEs

The data presented in Table 4 indicates the potential cancer risk (CR) associated with the consumption of pipe-borne water contaminated with chromium (Cr), cadmium (Cd), and lead (Pb) for different age groups (adults, children, and infants) across various locations. The mean CR values across the sampled locations for Cr ranged from 9.9E-04 ± 4.7E-04–1.7E-03 ± 1.4E-03 for adults, 1.5E-03 ± 7.4E-04–3.4E-03 ± 2.8E-03 for children, and 1.7E-03 ± 8.5E-04–3.8E-03 ± 3.2E-03 for infants. These values significantly exceed the permissible limit of 1.0 E-04, which corresponds to a risk of one in 10,000 individuals. The implications of this data suggest that approximately 10–17 adults, 15–34 children, and 17–38 infants out of every 10,000 individuals may be at risk of developing cancer from the consumption of these water samples.

**Table 4:** Cancer Risk Values of Metals in Pipe-borne water.

				Adults				Children				Infants	
		Mean	Std. Deviation	Minimum	Maximum	Mean	Std. Deviation	Minimum	Maximum	Mean	Std. Deviation	Minimum	Maximum
Cr	LAN	7.5E−04	3.8E−04	3.2E−04	1.4E−03	1.5E−03	7.5E−04	6.4E−04	2.9E−03	1.7E−03	8.5E−04	7.2E−04	3.2E−03
	ALO	9.9E−04	4.7E−04	3.2E−04	1.8E−03	2.0E−03	9.4E−04	6.4E−04	3.5E−03	2.2E−03	1.1E−03	7.2E−04	4.0E−03
	TEMI	1.7E−03	1.4E−03	1.6E−04	3.5E−03	3.4E−03	2.8E−03	3.2E−04	7.0E−03	3.8E−03	3.2E−03	3.6E−04	7.9E−03
	OLO	7.4E−04	4.5E−04	1.6E−04	1.6E−03	1.5E−03	9.1E−04	3.2E−04	3.2E−03	1.7E−03	1.0E−03	3.6E−04	3.6E−03
	GBON	1.6E−03	8.6E−04	4.8E−04	2.7E−03	3.2E−03	1.7E−03	9.6E−04	5.4E−03	3.6E−03	1.9E−03	1.1E−03	6.1E−03
	AJE	1.2E−03	1.5E−03	0.0E+ 00	5.1E−03	2.5E−03	3.1E−03	0.0E+ 00	1.0E−02	2.8E−03	3.5E−03	0.0E+ 00	1.2E−02
Cd	LAN	3.5E−03	2.0E−03	1.9E−03	7.8E−03	7.0E−03	4.0E−03	3.9E−03	1.6E−02	7.9E−03	4.5E−03	4.4E−03	1.8E−02
	ALO	4.5E−03	2.4E−03	1.9E−03	7.8E−03	9.0E−03	4.9E−03	3.9E−03	1.6E−02	1.0E−02	5.5E−03	4.4E−03	1.8E−02
	TEMI	3.1E−03	2.6E−03	0.0E+ 00	7.8E−03	6.2E−03	5.3E−03	0.0E+ 00	1.6E−02	7.0E−03	5.9E−03	0.0E+ 00	1.8E−02
	OLO	3.1E−03	2.8E−03	0.0E+ 00	7.8E−03	6.2E−03	5.6E−03	0.0E+ 00	1.6E−02	7.0E−03	6.3E−03	0.0E+ 00	1.8E−02
	GBON	5.3E−03	1.8E−03	1.9E−03	7.8E−03	1.1E−02	3.7E−03	3.9E−03	1.6E−02	1.2E−02	4.2E−03	4.4E−03	1.8E−02
	AJE	3.3E−03	2.6E−03	0.0E+ 00	7.8E−03	6.6E−03	5.2E−03	0.0E+ 00	1.6E−02	7.5E−03	5.9E−03	0.0E+ 00	1.8E−02
Pb	LAN	1.4E−04	4.3E−05	9.0E−05	2.1E−04	2.7E−04	8.5E−05	1.8E−04	4.1E−04	3.1E−04	9.6E−05	2.0E−04	4.6E−04
	ALO	1.6E−04	4.4E−05	1.0E−04	2.3E−04	3.3E−04	8.8E−05	2.1E−04	4.6E−04	3.7E−04	9.9E−05	2.3E−04	5.1E−04
	TEMI	1.5E−04	7.1E−05	5.4E−05	2.4E−04	3.0E−04	1.4E−04	1.1E−04	4.9E−04	3.4E−04	1.6E−04	1.2E−04	5.5E−04
	OLO	1.9E−04	7.1E−05	9.0E−05	2.9E−04	3.7E−04	1.4E−04	1.8E−04	5.7E−04	4.2E−04	1.6E−04	2.0E−04	6.4E−04
	GBON	1.5E−04	3.7E−05	8.4E−05	2.0E−04	3.0E−04	7.3E−05	1.7E−04	4.0E−04	3.4E−04	8.3E−05	1.9E−04	4.5E−04
	AJE	2.6E−04	1.2E−04	7.6E−05	4.2E−04	5.3E−04	2.5E−04	1.5E−04	8.3E−04	5.9E−04	2.8E−04	1.7E−04	9.4E−04

LAN- Lantoro, ALO- Alogi, TEMI- Temidire, OLO- Olorunsogo, GBON- Gbonagun, AJE- Ajegunle.

The average cancer risk (CR) observed for cadmium (Cd) ranged from 3.1E-03 ± 2.6E-03–5.3E-03 ± 1.8E-03 for adults, 6.2E-03 ± 5.3E-03–1.1E-02 ± 3.7E-03 for children, and 7.0E-03 ± 5.9E-03–1.2E-02 ± 4.2E-03 for infants.

These figures indicate that, in a population of 10,000, approximately 31–53 adults, 62–110 children, and 70–120 infants may have an increased risk of developing cancer from consuming contaminated water samples.

Lead (Pb) indicated mean cancer risk (CR) values ranging from 1.4E-04 ± 4.3E-05–2.6E-04 ± 4.3E-05 for adults, 2.7E-04 ± 8.5E-05–5.3E-04 ± 2.5E-04 for children, and 3.1E-04 ± 9.6E-05–5.9E-04 ± 2.8E-04 for infants.

These data suggest that approximately 1–3 adults, 3–5 children, and 3–6 infants in a population of 10,000 may have an increased propensity to develop cancer from the consumption of contaminated water samples.

Cadmium (Cd) generally presents the highest mean cancer risk values across all locations and age groups, followed by another unspecified contaminant, while lead (Pb) has the lowest cancer risk values. Infants consistently exhibit the highest cancer risk values for all contaminants across all locations. A previous study has shown CR exceeding the permissible limit of 1E-04 (i.e., 1 out of 10,000 populations) for Cd, Cr and Pb in drinkable groundwater from Chandigarh, India [28].

**Fig. 3:**
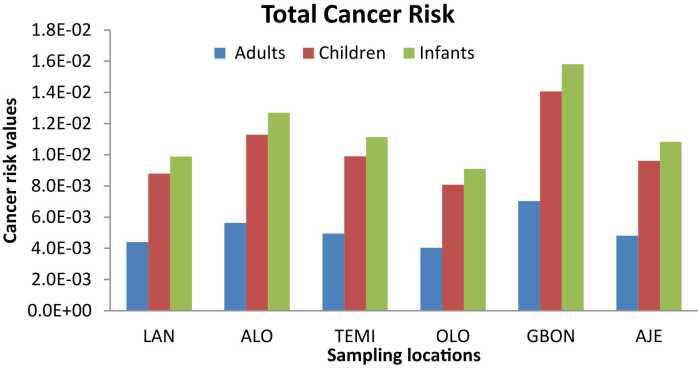
Sum of Cancer Risk Values of Metals in Pipe-borne water. LAN- Lantoro, ALO- Alogi, TEMI- Temidire, OLO- Olorunsogo, GBON- Gbonagun, AJE- Ajegunle.

Fig. 3 illustrates the total cancer risk (CR) values of potential toxic metals (PTEs) at various sampling locations for different population groups, including adults, children, and infants. The total CR values exceeded the permissible limit of 1E-04 across all categories of the exposed population, indicating a potential risk for cancer development.

**Fig. 4:**
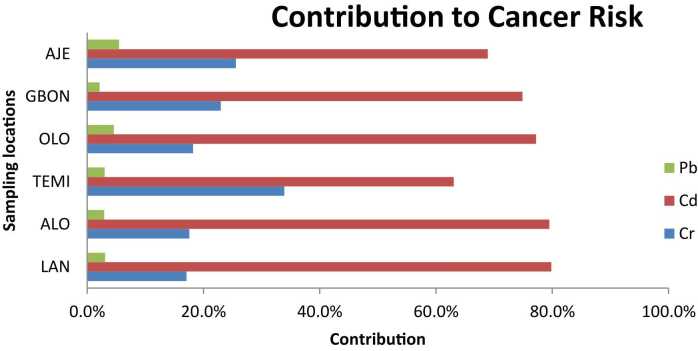
Contributions by Metals to Cancer Risk, LAN- Lantoro, ALO- Alogi, TEMI- Temidire, OLO- Olorunsogo, GBON- Gbonagun, AJE- Ajegunle.

Fig. 4 presents the contribution of various metals to the total cancer risk (TCR). Cadmium (Cd) emerged as the predominant contributor across all six communities, accounting for 63–79 % of the TCR. Chromium (Cr) was the second-largest contributor, with its contribution ranging from 17 % to 33 %. Notably, hexavalent chromium is a well-documented carcinogen. Lead (Pb) contributed the least to the total cancer risk, accounting for only 2–5 % across all locations.

### 3.3 Water quality index of potentially toxic elements

**Fig. 5:**
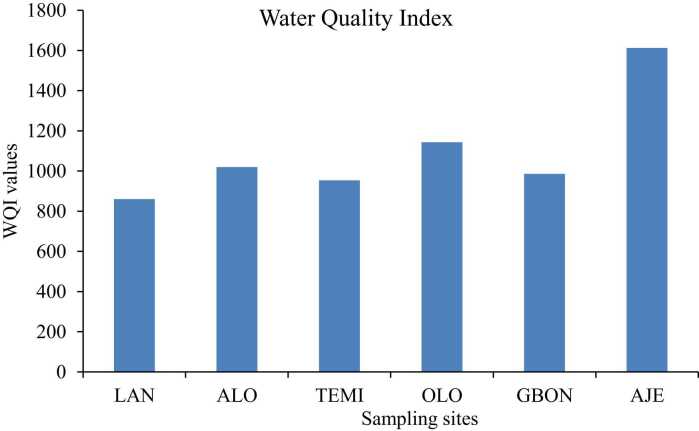
Water quality index of PTEs in pipe-borne water, LAN- Lantoro, ALO- Alogi, TEMI- Temidire, OLO- Olorunsogo, GBON- Gbonagun, AJE- Ajegunle.

Fig. 5 shows the Water Quality Index (WQI) values of potentially toxic elements (PTEs) in pipe-borne water samples from the study area. The WQI values for PTEs ranged from 861 (Lantoro) to 1613 (Ajegunle). At each location within the study area, the WQI values exceeded the maximum threshold limit of 300, indicating that the water is unfit for consumption [27]. Lead constituted the largest portion of the WQI, accounting for 87–94 % of the total WQI values at all monitoring sites. A study by Yilkal et al. [43] demonstrated that the WQI values in bottled water marketed in Addis Ababa, Ethiopia, were less than 100. In contrast, the WQI values of sachet water collected from various parts of Abeokuta metropolis were generally below 100, indicating good quality.

## 4 Conclusion

This study assessed the levels of potentially toxic elements (PTEs) in pipe-borne water collected from sixty households in Abeokuta Metropolis, Ogun State, Nigeria. All the physicochemical parameters observed in the pipe-borne water were within the permissible limits set by the World Health Organization (WHO). However, elevated levels of PTEs such as cadmium (Cd), chromium (Cr), and lead (Pb) were detected in most water samples, exceeding the WHO standards for drinking water.

The non-carcinogenic adverse health effects associated with Cr, Cd, and Pb were identified, with hazard quotient values greater than the permissible limit of 1.0. Similarly, the cancer risk for Cd, Cr, and Pb also surpassed the safe threshold limit of 1.0 × 10^−4^, indicating potential carcinogenic effects. These findings underscore the urgent need for stringent regulatory measures and regular monitoring to ensure water safety in the study area. There is a critical need for improved water treatment processes, ongoing monitoring, and public awareness initiatives to mitigate these risks. Removal of potential toxic elements at the point of collection by using taps fitted with activated carbon filters is also suggested. Furthermore, replacement or lining of pipes to mitigate corrosion is recommended.

## Limitation of the Study

A significant limitation of this study is the sampling frequency; therefore, future monitoring for a minimum of three months is suggested.

## Declaration of Competing Interest

The authors declare that they have no known competing financial interests or personal relationships that could have appeared to influence the work reported in this paper

## Data Availability

Data included in manuscript and supplementary informatiom
