# Supplementary material for: Health risk assessment of potentially toxic elements in public pipe-borne water from selected households in Abeokuta, Southwestern, Nigeria
Source: Toxicol Rep. 2025 Feb 20;14:101970. doi: 10.1016/j.toxrep.2025.101970 (PMC11891697; doi:10.1016/j.toxrep.2025.101970)
Supplement: Supplementary file 1 — Supplementary material [file mmc1.docx]

**HEALTH RISK ASSESSMENT OF POTENTIALLY TOXIC ELEMENTS IN PUBLIC PIPE-BORNE WATER FROM SELECTED HOUSEHOLDS IN ABEOKUTA, SOUTHWESTERN, NIGERIA**

**Adewale M. Taiwo* and Adediran O. Coker**

**Department of Environmental Management and Toxicology, Federal University of Agriculture, PMB 2240, Abeokuta, Ogun State, Nigeria**

**Supplementary Information**

Table S1: Detailed information regarding the WQI of these PTEs

| Site C | | | | S1 | w | W_n_ | Q1 | W_n_ × Q1 |
| --- | --- | --- | --- | --- | --- | --- | --- | --- |
|  | Zn | LAN | 0.23 | 3.00 | 3 | 0.09375 | 11.67 | 1.09 |
|  | (mg/L) | ALO | 0.35 |  |  |  | 9.00 | 0.84 |
|  |  | TEMI | 0.27 |  |  |  | 8.67 | 0.81 |
|  |  | OLO | 0.26 |  |  |  | 21.67 | 2.03 |
|  |  | GBON | 0.65 |  |  |  | 16.67 | 1.56 |
|  |  | AJE | 0.5 |  |  |  | 1.00 | 0.09 |
|  | Cu | LAN | 0.03 | 2 | 1 | 0.03125 | 1.50 | 0.05 |
|  |  | ALO | 0.05 |  |  |  | 2.50 | 0.08 |
|  | (mg/L) | TEMI | 0.06 |  |  |  | 3.00 | 0.09 |
|  |  | OLO | 0.07 |  |  |  | 3.50 | 0.11 |
|  |  | GBON | 0.13 |  |  |  | 6.50 | 0.20 |
|  |  | AJE | 0.12 |  |  |  | 6.00 | 0.19 |
|  | \| Cr \| \| --- \| | LAN | 0.05 | 0.05 | 5 | 0.15625 | 100.00 | 15.63 |
|  | (mg/L) | ALO | 0.06 |  |  |  | 120.00 | 18.75 |
|  |  | TEMI | 0.1 |  |  |  | 200.00 | 31.25 |
|  |  | OLO | 0.05 |  |  |  | 100.00 | 15.63 |
|  |  | GBON | 0.1 |  |  |  | 200.00 | 31.25 |
|  |  | AJE | 0.11 |  |  |  | 220.00 | 34.38 |
|  | Cd | LAN | 0.02 | 0.05 | 5 | 0.15625 | 40.00 | 6.25 |
|  |  | ALO | 0.02 |  |  |  | 40.00 | 6.25 |
|  | (mg/L) | TEMI | 0.02 |  |  |  | 40.00 | 6.25 |
|  |  | OLO | 0.02 |  |  |  | 40.00 | 6.25 |
|  |  | GBON | 0.03 |  |  |  | 60.00 | 9.38 |
|  |  | AJE | 0.02 |  |  |  | 40.00 | 6.25 |
|  | Pb | LAN | 0.5 | 0.01 | 5 | 0.15625 | 5000.00 | 781.25 |
|  |  | ALO | 0.6 |  |  |  | 6000.00 | 937.50 |
|  | (mg/L) | TEMI | 0.55 |  |  |  | 5500.00 | 859.38 |
|  |  | OLO | 0.68 |  |  |  | 6800.00 | 1062.50 |
|  |  | GBON | 0.55 |  |  |  | 5500.00 | 859.38 |
|  |  | AJE | 0.97 |  |  |  | 9700.00 | 1515.63 |

LAN- Lantoro, ALO- Alogi, TEMI- Temidire, OLO- Olorunsogo, GBON- Gbonagun, AJE- Ajegunle., SI_n_ = sub-index of n^th^ parameter, Q_n_ = Water quality rating scale, and W_n_ = Relative weight.

Table S2: Estimated Daily Dose of Metals in Groundwater Ingested By Infants, Children and Adults

|  |  |  |  | Adults |  |  |  | Children |  |  |  | Infants |  |
| --- | --- | --- | --- | --- | --- | --- | --- | --- | --- | --- | --- | --- | --- |
|  |  | Mean | Std. Deviation | Minimum | Maximum | Mean | Std. Deviation | Minimum | Maximum | Mean | Std. Deviation | Minimum | Maximum |
| Zn | LAN | 5.1E-03 | 6.3E-03 | 0.0E+00 | 2.0E-02 | 1.0E-02 | 1.3E-02 | 0.0E+00 | 4.0E-02 | 1.1E-02 | 1.4E-02 | 0.0E+00 | 4.5E-02 |
|  | ALO | 7.8E-03 | 7.7E-03 | 0.0E+00 | 2.5E-02 | 1.6E-02 | 1.5E-02 | 0.0E+00 | 4.9E-02 | 1.8E-02 | 1.7E-02 | 0.0E+00 | 5.5E-02 |
|  | TEMI | 7.0E-03 | 6.5E-03 | 0.0E+00 | 2.2E-02 | 1.4E-02 | 1.3E-02 | 0.0E+00 | 4.4E-02 | 1.6E-02 | 1.5E-02 | 0.0E+00 | 5.0E-02 |
|  | OLO | 6.7E-03 | 7.2E-03 | 0.0E+00 | 2.0E-02 | 1.3E-02 | 1.4E-02 | 0.0E+00 | 4.0E-02 | 1.5E-02 | 1.6E-02 | 0.0E+00 | 4.5E-02 |
|  | GBON | 2.1E-02 | 1.1E-02 | 8.0E-03 | 3.8E-02 | 4.2E-02 | 2.2E-02 | 1.6E-02 | 7.7E-02 | 4.7E-02 | 2.4E-02 | 1.8E-02 | 8.6E-02 |
|  | AJE | 1.6E-02 | 8.4E-03 | 5.4E-03 | 3.1E-02 | 3.2E-02 | 1.7E-02 | 1.1E-02 | 6.1E-02 | 3.6E-02 | 1.9E-02 | 1.2E-02 | 6.9E-02 |
| Cu | LAN | 8.6E-04 | 1.0E-03 | 0.0E+00 | 2.9E-03 | 1.7E-03 | 2.1E-03 | 0.0E+00 | 5.8E-03 | 1.9E-03 | 2.4E-03 | 0.0E+00 | 6.5E-03 |
|  | ALO | 1.4E-03 | 1.8E-03 | 0.0E+00 | 4.5E-03 | 2.8E-03 | 3.5E-03 | 0.0E+00 | 8.9E-03 | 3.2E-03 | 4.0E-03 | 0.0E+00 | 1.0E-02 |
|  | TEMI | 1.6E-03 | 1.7E-03 | 0.0E+00 | 5.8E-03 | 3.1E-03 | 3.4E-03 | 0.0E+00 | 1.2E-02 | 3.5E-03 | 3.8E-03 | 0.0E+00 | 1.3E-02 |
|  | OLO | 2.3E-03 | 1.7E-03 | 3.2E-04 | 5.8E-03 | 4.7E-03 | 3.4E-03 | 6.4E-04 | 1.2E-02 | 5.2E-03 | 3.8E-03 | 7.2E-04 | 1.3E-02 |
|  | GBON | 4.1E-03 | 2.0E-03 | 1.3E-03 | 6.7E-03 | 8.2E-03 | 4.0E-03 | 2.6E-03 | 1.3E-02 | 9.2E-03 | 4.5E-03 | 2.9E-03 | 1.5E-02 |
|  | AJE | 3.7E-03 | 2.3E-03 | 1.3E-03 | 8.9E-03 | 7.4E-03 | 4.6E-03 | 2.6E-03 | 1.8E-02 | 8.3E-03 | 5.1E-03 | 2.9E-03 | 2.0E-02 |
| Cr | LAN | 1.5E-03 | 7.5E-04 | 6.4E-04 | 2.9E-03 | 3.0E-03 | 1.5E-03 | 1.3E-03 | 5.8E-03 | 3.4E-03 | 1.7E-03 | 1.4E-03 | 6.5E-03 |
|  | ALO | 2.0E-03 | 9.4E-04 | 6.4E-04 | 3.5E-03 | 4.0E-03 | 1.9E-03 | 1.3E-03 | 7.0E-03 | 4.5E-03 | 2.1E-03 | 1.4E-03 | 7.9E-03 |
|  | TEMI | 3.4E-03 | 2.8E-03 | 3.2E-04 | 7.0E-03 | 6.7E-03 | 5.7E-03 | 6.4E-04 | 1.4E-02 | 7.6E-03 | 6.4E-03 | 7.2E-04 | 1.6E-02 |
|  | OLO | 1.5E-03 | 9.1E-04 | 3.2E-04 | 3.2E-03 | 2.9E-03 | 1.8E-03 | 6.4E-04 | 6.4E-03 | 3.3E-03 | 2.0E-03 | 7.2E-04 | 7.2E-03 |
|  | GBON | 3.2E-03 | 1.7E-03 | 9.6E-04 | 5.4E-03 | 6.5E-03 | 3.4E-03 | 1.9E-03 | 1.1E-02 | 7.3E-03 | 3.9E-03 | 2.2E-03 | 1.2E-02 |
|  | AJE | 2.5E-03 | 3.1E-03 | 0.0E+00 | 1.0E-02 | 4.9E-03 | 6.2E-03 | 0.0E+00 | 2.0E-02 | 5.5E-03 | 7.0E-03 | 0.0E+00 | 2.3E-02 |
| Cd | LAN | 5.8E-04 | 3.3E-04 | 3.2E-04 | 1.3E-03 | 1.2E-03 | 6.6E-04 | 6.4E-04 | 2.6E-03 | 1.3E-03 | 7.4E-04 | 7.2E-04 | 2.9E-03 |
|  | ALO | 7.4E-04 | 4.0E-04 | 3.2E-04 | 1.3E-03 | 1.5E-03 | 8.0E-04 | 6.4E-04 | 2.6E-03 | 1.7E-03 | 9.0E-04 | 7.2E-04 | 2.9E-03 |
|  | TEMI | 5.1E-04 | 4.3E-04 | 0.0E+00 | 1.3E-03 | 1.0E-03 | 8.6E-04 | 0.0E+00 | 2.6E-03 | 1.2E-03 | 9.7E-04 | 0.0E+00 | 2.9E-03 |
|  | OLO | 5.1E-04 | 4.6E-04 | 0.0E+00 | 1.3E-03 | 1.0E-03 | 9.1E-04 | 0.0E+00 | 2.6E-03 | 1.2E-03 | 1.0E-03 | 0.0E+00 | 2.9E-03 |
|  | GBON | 8.6E-04 | 3.0E-04 | 3.2E-04 | 1.3E-03 | 1.7E-03 | 6.1E-04 | 6.4E-04 | 2.6E-03 | 1.9E-03 | 6.8E-04 | 7.2E-04 | 2.9E-03 |
|  | AJE | 5.4E-04 | 4.3E-04 | 0.0E+00 | 1.3E-03 | 1.1E-03 | 8.6E-04 | 0.0E+00 | 2.6E-03 | 1.2E-03 | 9.6E-04 | 0.0E+00 | 2.9E-03 |
| Pb | LAN | 1.6E-02 | 5.0E-03 | 1.1E-02 | 2.4E-02 | 3.2E-02 | 1.0E-02 | 2.1E-02 | 4.9E-02 | 3.6E-02 | 1.1E-02 | 2.4E-02 | 5.5E-02 |
|  | ALO | 1.9E-02 | 5.2E-03 | 1.2E-02 | 2.7E-02 | 3.9E-02 | 1.0E-02 | 2.4E-02 | 5.4E-02 | 4.3E-02 | 1.2E-02 | 2.7E-02 | 6.0E-02 |
|  | TEMI | 1.8E-02 | 8.3E-03 | 6.4E-03 | 2.9E-02 | 3.5E-02 | 1.7E-02 | 1.3E-02 | 5.8E-02 | 4.0E-02 | 1.9E-02 | 1.4E-02 | 6.5E-02 |
|  | OLO | 2.2E-02 | 8.4E-03 | 1.1E-02 | 3.4E-02 | 4.4E-02 | 1.7E-02 | 2.1E-02 | 6.7E-02 | 4.9E-02 | 1.9E-02 | 2.4E-02 | 7.6E-02 |
|  | GBON | 1.8E-02 | 4.3E-03 | 9.9E-03 | 2.4E-02 | 3.5E-02 | 8.6E-03 | 2.0E-02 | 4.7E-02 | 4.0E-02 | 9.7E-03 | 2.2E-02 | 5.3E-02 |
|  | AJE | 3.1E-02 | 1.5E-02 | 8.9E-03 | 4.9E-02 | 6.2E-02 | 2.9E-02 | 1.8E-02 | 9.8E-02 | 7.0E-02 | 3.3E-02 | 2.0E-02 | 1.1E-01 |

LAN- Lantoro, ALO- Alogi, TEMI- Temidire, OLO- Olorunsogo, GBON- Gbonagun, AJE- Ajegunle.
